# “Not just the consequences, but also the pleasurable sex”: a review of the content of comprehensive sexuality education for early adolescents in Rwanda

**DOI:** 10.1186/s12889-022-14966-0

**Published:** 2023-01-07

**Authors:** Valens Mbarushimana, Susan Goldstein, Daphney Nozizwe Conco

**Affiliations:** 1 School of Public Health, Faculty of Health Sciences, University of the Witwatersrand, Johannesburg, South Africa; 2 School of Public Health, College of Medicine and Health Sciences, University of Rwanda, Kigali, Rwanda; 3 SAMRC/Centre for Health Economics and Decision Science, School of Public Health, Faculty of Health Sciences, PRICELESS SA, University of the Witwatersrand, Johannesburg, South Africa

**Keywords:** Early adolescent, Sexual and reproductive health and rights, Comprehensive sexuality education, Sexually transmitted infection, Gender, Competence-based curriculum, ITGSE, Rwanda

## Abstract

**Background:**

Responding to adolescents’ educational needs in sexual and reproductive health and rights (SRHR) is central to their sexual health and achieved through school-based comprehensive sexuality education (CSE). In 2016, Rwanda introduced CSE through the competence-based curriculum in schools to enhance learners’ knowledge about sexuality, gender, and reproductive health issues, including HIV/AIDS. However, globally, the content of CSE is sometimes dissimilar, and little evidence surrounds its scope in many settings, including Rwanda. In addition, the extent to which CSE aligns with international guidelines has yet to be well known. This study assesses major areas of CSE for early adolescents in Rwanda, analyses how CSE correlates with international guidelines and makes recommendations accordingly.

**Methods:**

We reviewed the Rwandan competence-based curriculum to map CSE competences for early adolescents and conducted semi-structured interviews with key informants (N = 16). Eleven of the 23 curriculum documents met the selection criteria and were included in the final review. We manually extracted data using a standard form in Microsoft Excel and analysed data using frequency tables and charts. Interviews were thematically analysed in NVivo 11 for Windows.

**Findings:**

We found 58 CSE competences for early adolescents across various subjects, increasing with school grades. All recommended CSE areas were addressed but to a variable extent. Most competences fall under four recommended areas: sexual and reproductive health; human body and development; values, rights, and sexuality; and understanding gender. The least represented area is violence and staying safe. Of the 27 expected topics, there are two to six CSE competences for 13 topics, one CSE competence for each of the six others, and none for the eight remaining ones. Qualitative findings support these findings and suggest additional content on locally controversial but recommended areas of sexual pleasure, orientation, desire and modern contraceptive methods.

**Conclusion:**

This study explores the CSE content for early adolescents in Rwanda and how they align with sexuality education standards. Ensuring equal coverage of CSE areas and addressing missing topics may improve CSE content for this age group and foster their SRHR.

## Background

Many countries around the globe have a growing interest in ensuring that children and young people have access to some form of sexuality education [1]. The 1994 international conference on population and development (ICPD)’s Programme of Action highlighted the role of Governments in promoting adolescent sexual and reproductive health and rights through delivering sexuality education [2, 3]. Whether offered in formal (school) or non-formal (community) settings, the ICPD specified that sexuality education should be age-appropriate, start as early as possible, and foster mature decision-making [2]. Comprehensive Sexuality Education (CSE) is an important way to promote adolescent sexual and reproductive health and rights [4–6] and becomes crucial during early adolescence (10–14 years), a stage of transition from childhood to older adolescence and adulthood, laying the ground for their future sexual and reproductive health (SRH), gendered attitudes, and behaviours [7, 8].

There is evidence that adolescents benefit from sexuality education programmes in many ways. For example, a sample of students from three departments in Guatemala indicated that most students (89–96%) reported personal benefits from CSE in 2015 [9]. Evidence also suggests that sexuality education programmes improved young people’s SRHR knowledge, attitude and behaviour in Nigeria [10, 11], Ethiopia [12] and Korea [13]. While examining the effects of school sexuality education programmes on adolescents’ sexual knowledge, Song et al. [14] concluded that sexuality education positively affects overall sexual knowledge. Specifically, CSE positively impacts sexual knowledge, attitudes, communication, and sexual behaviours among adolescents [15, 16]. CSE is an effective tool to ensure that adolescents access the SRH information they need to make healthy and informed Sexual and Reproductive Health and Rights (SRHR) choices. It enables adolescents to discuss sex, know scientific facts, and have social skills to use this knowledge to resist peer pressure [17]. In addition, CSE improves the perception of SRH risks and knowledge of early adolescents [18], delays sex initiation and decreases adolescent pregnancy [19]. Furthermore, a systematic review and meta-analysis in low- and middle-income countries revealed that CSE contributed to safe sexual health practices such as condom use, refusing sex, reduced sexual partners and initiation of first sex [20]. On the other hand, some studies report that sexuality education is a motive to initiate sexual behaviours [21] though this is not a commonly held belief among CSE practitioners and researchers.

The content of CSE remains contested in many parts of the world due to variations in framing and notions of sexuality, fears related to disrupting identities, values of the communities and compromising children’s innocence [5]. In addition, a literature review of CSE guidelines indicates that there is no agreed upon definition of the term “comprehensive” in the context of CSE [4, 22]. Because of these varied understandings and fears about CSE, several sexuality education guidelines have been developed [23–27]. These guidelines emanate from the Sexuality Information and Education Council of the United States (SIECUS) [26]; the World Health Organization Europe and Federal Centre for Health Education BZgA [25]; and UNESCO [24]. Of these guidelines, UNESCO’s International Technical Guidance on Sexuality Education (ITGSE) offers many advantages because it has contents for all learners’ grades and provides a research evidence-based framework for use by those interested in developing CSE programs [6, 28]. The ITGSE outlines eight key concepts as major components of a CSE curriculum [6, 24]. These key concepts include (1) relationships; (2) values, rights, culture, and sexuality; (3 )understanding gender; (4) violence and staying safe; (5) skills for health and wellbeing; (6) the human body and development; (7) sexuality and sexual behaviour; and (8) sexual and reproductive health [24]. Each of these key concepts highlights important topics of interest for sexuality education depending on the age of the learners. The ITGSE organises CSE into four age groups 5–8 years, 9–12 years, 12–15 years, and 15–18 + years [24].

Despite the perceived importance of delivering accurate SRHR information to adolescents, there is limited evidence of CSE coverage. Studies highlight that the implementation of CSE programs may not be optimal, and the content is dissimilar in many parts of the world. For example, in Sub-Saharan Africa, religious communities exclude some CSE topics such as masturbation, abortion and sexual orientation because they contradict their commonly held beliefs [29]. In addition, some communities resist teaching about gender equality fearing the disruption of patriarchal system that maintains men dominant and women compliant [29]. A review of the CSE curriculum in East and Southern Africa revealed gaps in information about condoms, contraception and other SRH topics. Attention to gender content was weak or contradictory [2, 3]. Haberland and Rogow [2] noted that effective CSE should engage a gender and rights perspective. A study of the quality of sexuality education implementation in low- and middle-income countries found that CSE focused more on contraception and unintended pregnancy than other topics and emphasised more factual knowledge than skill-based topics [30].

In 2016, the Rwanda Education Board introduced the competence-based curriculum integrating CSE at the basic education level [31, 32], to equip young people, including early adolescents, with knowledge, skills, and values that allow them to decide responsibly “about their sexual and social relationships, explain and clarify feelings, values, and attitudes, and promote and sustain risk-reducing behaviours” [31]. The Rwandan curriculum framework specifies that a competence-based curriculum aims to improve the learners’ experience by focusing more on their competence than their knowledge [31], and a competence is defined as the ability to combine “knowledge, skills, attitudes, values and behaviour to accomplish a particular task” [33]. Furthermore, this curriculum framework indicates that prior to introducing CSE in schools, only a small proportion of Rwandan learners received information about their transition from childhood to adolescence and adulthood and healthy sexual lives [33]. Consequently, the Government of Rwanda introduced CSE to respond to inadequate knowledge of sexuality, gender and other reproductive health issues such as HIV/AIDS [33]. In addition, 1,508 teachers and other education stakeholders were trained on key features of this curriculum integrating CSE; and a teacher’s guide on CSE for secondary schools was published in 2018 [34]. The competence-based curriculum indicates that CSE is a cross-cutting issue taught across different designated subjects [31]. Cross-cutting issues have connection with different subject areas and are important to learn about, are not confined to one subject, and integrated across years and cycles [31].

Since the introduction of this curriculum into schools, no studies have been conducted to assess the major areas addressed by the curriculum mainly for early adolescents to understand the focus of CSE and address potential gaps for this age group. In addition, there is limited evidence on how the Rwandan competence-based curriculum integrates the ITGSE recommendations. Based on ITGSE [24], this study aims to assess major areas of CSE for early adolescents in Rwandan schools as reflected in the competence-based curriculum, analyse the level to which this curriculum addresses various recommended key concepts and topics from the ITGSE and suggest areas of improvement.

## Methods

### Study setting

We conducted this study in Rwanda, where the basic education system consists of pre-primary, primary, and secondary education [31]. Pre-primary education comprises nursery schools and enrols learners between three and six years. Primary education lasts for six years, consists of lower primary (P1-P3) and upper primary (P4-P6) and hosts learners aged six to 12 years. Secondary education lasts for six years and is organised into the lower or ordinary level (O-Level) and upper or advanced level (A-Level). Secondary education officially enrols learners between 13 and 18 years; however, some children start secondary schooling at 12 years. The languages of teaching and learning are Kinyarwanda for pre-primary and lower primary (P1-P3) levels and English for upper primary (P4-P6) and secondary levels [31]. Teaching programs, including the competence-based curriculum for these levels, are developed and monitored by the Rwanda Education Board (REB) [35].

The Rwanda competence-based curriculum specifies that learning takes place in different subjects at the pre-primary, primary and lower-secondary levels [36]. At the pre-primary level, learning activities take place in areas of Discovery of the World, Numeracy, Physical and Health Development, Creative Arts and Culture; Language and Literacy: (Kinyarwanda and English), and Social and Emotional Development. At the lower-primary levels, learning takes place in the following core subjects: Kinyarwanda, English, Mathematics, Social and Religious Studies, Science and Elementary Technology, Creative Arts: Music, Fine Art and Craft, Physical education. Core subjects for the upper-primary level are the same as those listed for the lower-primary level, in addition to the subject of French. The core subjects of Kinyarwanda, English, Mathematics, French, Physical education (and Sports) are also opened at the upper-secondary level in addition to the following ones: Physics, Chemistry, Biology and Health Sciences, Information and Communication Technology, History and Citizenship, Geography and Environment, Entrepreneurship, Kiswahili, Literature in English. at the lower-secondary level, the learners have Religion and Ethics, Music, Dance and Drama, Fine arts and Crafts, Home Sciences, Farming (Agriculture and Animal husbandry) as elective subjects and Library and Clubs as compulsory co-curricular activity.

Religious institutions play an important role at many levels of the Rwanda’s education system. The fourth population and housing census conducted in 2012 indicated that religious affiliation was universal with the predominance of Christianity [37]. In 2018, education statistics in Rwanda indicate that religious institutions own most of the pre-primary, primary and secondary schools [38]. In Rwanda, cultural and religious influences are perceived as barriers to delivering sexual and reproductive health services related to family planning and circumcision [39].

### Study design

This mixed-method study involved a review of the Rwanda competence-based curriculum CSE and key informant interviews. We reviewed syllabi of subjects accommodating the CSE curriculum in the Rwanda curriculum for the pre-primary, primary, and lower secondary school levels to map the sexuality education competences. The review followed the revised sexuality education framework provided by UNESCO [24]. In addition, key informant interviews were conducted to explore informants’ perceptions about the focus of sexuality education for Rwandan early adolescents and potential areas of improvement.

### Study participants

We officially contacted the institutions of key informants to select potential study participants. Selected were key informants who were regularly involved in the sexuality education of early adolescents in Rwanda. After identifying and selecting the participants, the researcher and participants agreed upon further study arrangements and interviews appointments.

We purposively selected the key informants (eight male and eight female) based on their positions as policy-makers, religious leaders, and private stakeholders working in the field of adolescent sexual and reproductive health in Rwanda. The semi-structured interviews involved in total 16 key informants, of whom nine participants (three males and six females) belonged to public institutions such as Ministries (4), Government-affiliated agencies (3), and District level institutions (2). There were also six key informants (five male and one female) from Faith-based Organisations (FBOs) and one female participant from a Non-Governmental Organization.

### Selection of documents for review

The document review process entailed searching for CSE competences from the Rwanda competence-based curriculum and classifying these competences per the key concepts and topics recommended in the UNESCO’s ITGSE. The selection process involved identifying the subjects designated by the competence-based curriculum to integrate CSE at the pre-primary, primary (P1-P6), and lower secondary (S1-S3) levels [36]. There were 13 identified subjects: (1) Social Studies, (2) History and Citizenship, (3) General Studies, (4) English, (5) French, (6) Kinyarwanda, (7) Kiswahili, (8) Information and communication Technology, (9) Music, (10) Physical Education, (11) Biology, (12) Science and Elementary Technology, and (13) Religious Education [33, 40]. For the 13 subjects, there were 23 syllabi following the school levels [33], which included: one syllabus for the pre-primary level; five syllabi for the lower-primary (P1-P3) level; six syllabi for the upper-primary (P4-P6) level; and 11 syllabi for the lower-secondary (S1-S3) level. However, of the 23 syllabi of the subjects indicated by the Rwandan competence-based curriculum to accommodate CSE [31], only 11 syllabi were found to have CSE content, and these were selected for the final review (Table 1).

### Data collection

We collected data using two methods: the record review and the key informant interviews. The record review involved searching for CSE competencies from the selected syllabi. We used search terms including gender, sex, sexuality, sexual activity, sexually transmitted infections, HIV/AIDS, values, rights, culture, violence and staying safe, skills for health and wellbeing, human body and development, sexual behaviour, and reproductive health. We mapped key terms from the competence-based curriculum’s syllabi, assessed, linked them to specific CSE competences defined in this curriculum and extracted data in a Microsoft Excel form for further analysis. The data extraction form consisted of the following columns: document title, school grade, subject, topic, competence, and suggested contents.

The interviews with key informants explored their views about the major areas of CSE for early adolescents in Rwanda, and the content that should be part of the school curriculum. Specifically, the interview outline focused mainly on what the participants think early adolescents learn about gender and sexuality in the school curriculum and what they think these early adolescents should learn (appropriate content for this age group) from this curriculum. The study did not aim to ask participants about the factors related to the implementation of CSE in Rwanda, and how this implementation influences learning. The interviews took place per appointment and at a place indicated by the participants, mainly in their offices. Except for one interview, we conducted all the interviews in Kinyarwanda, the principal language of the informants. First, we transcribed verbatim voice records of interviews, and then professional translators translated these interview transcripts from Kinyarwanda to English. Finally, we compared the translated text to the original version to ensure similarity [41].

### Data analysis

We assessed the CSE competencies identified from the Rwanda competence-based curriculum and categorised these CSE competences per ITGSE concepts and topics [24]. These key concepts include (1) Relationships; (2) Values, Rights, Culture and Sexuality; (3) Understanding Gender; (4) Violence and Staying Safe; (5) Skills for Health and Well-being; (6) Human Body and Development; (7) Sexuality and sexual behaviour; and (8) Sexual and Reproductive Health [24]. Data from the curriculum review were summarized using frequency tables and charts and organised data per subject, school grade, concepts and topics of ITGSE. In addition, we imported key informant interview data into NVivo for Windows 11 (QSR International Pty) for thematic analysis using the ITGSE framework. Finally, we used quotations to support the description of qualitative data from key informant interviews.

## Results

This section provides an overview of CSE competences from the competence-based curriculum per school grades and recommended areas of CSE. It also highlights participants’ perceptions on major areas of CSE and their recommendations regarding the content of CSE for early adolescents in Rwanda.

### CSE competences per school grade

The findings showed that teaching CSE starts in the pre-primary level and the number of CSE competences in the competence-based curriculum increased with higher school grades. Overall, there were 58 CSE competences from the pre-primary to the lower-secondary levels. There were only four CSE competences at the pre-primary level, 22 competences at the primary level, and 32 competences at the lower-secondary level. Overall, upper-primary and lower-secondary levels accounted for most CSE competences (49/58) (Table 1).

**Table 1:** Distribution of CSE competences per school grade

	Number of CSE Competences	Percent
**School grade**		
Preschool 1 (3–4 years)	2	3.4
Preschool 2 (4–5 years)	1	1.7
Preschool 3 (5–6 years)	1	1.7
**Pre-primary (Preschool 1–3)**	**4**	**6.9**
Primary 1	2	3.4
Primary 2	1	1.7
Primary 3	2	3.4
**Lower primary (Primary 1–3)**	**5**	**8.6**
Primary 4	2	3.4
Primary 5	5	8.6
Primary 6	10	17.2
**Upper primary (Primary 4–6)**	**17**	**29.3**
Secondary 1	10	17.2
Secondary 2	11	19.0
Secondary 3	11	19.0
**Lower secondary (Secondary 1–3)**	**32**	**55.2**
**Total**	**58**	**100.0**

### Areas of CSE for early adolescents in Rwanda

**Fig. 1:**
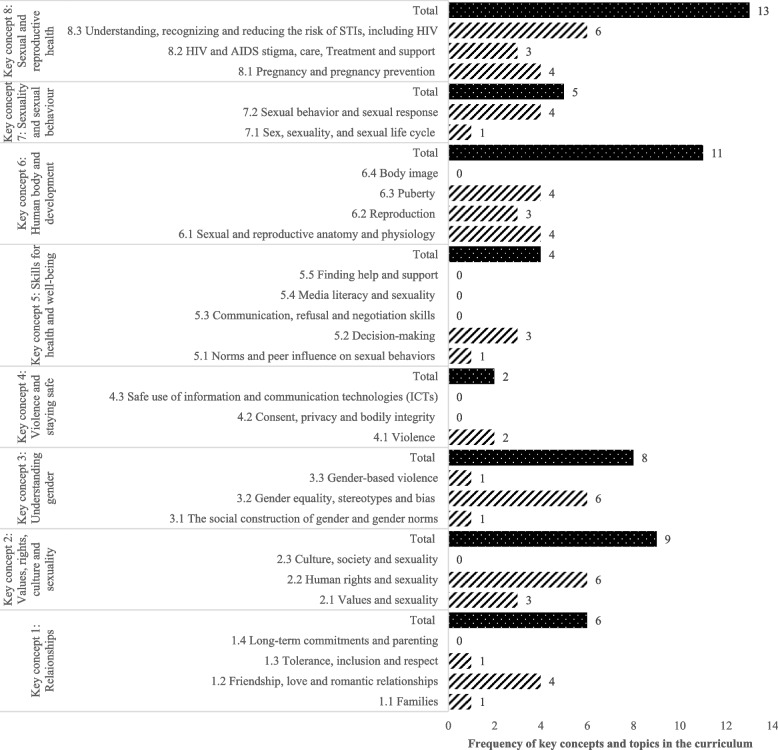
Distribution of CSE competences for early adolescents in Rwanda per standard areas of sexuality education

The distribution of CSE competences across various recommended areas indicated that the most addressed area was sexual and reproductive health. This area is organised under three topics: pregnancy and pregnancy prevention; HIV and AIDS stigma, care, treatment and support; and understanding, recognising and reducing the risk of STIs, including HIV [24]. There were 13 CSE competences in the curriculum for this area, six of which fell under the topic of understanding, recognising, and reducing the risk of sexually transmitted infections, including HIV (Fig. 1). According to the qualitative findings related to this area, participants perceived that CSE focuses more on pregnancy prevention for girls because the consequences of pregnancy are more severe for them than for boys, and they do not conceive. Participants indicated that education of early adolescents focuses on pregnancy prevention methods, including condoms and other contraceptive methods. In addition, the participants noted that preventing sexually transmitted infections such as HIV/AIDS, hepatitis, and cervical cancer was another focus of sexuality education for early adolescents. The participants also pointed out that circumcision was becoming an area of growing interest in CSE of early adolescents. Finally, they noted that such education focused on available services to support early adolescents in sexual and reproductive health matters. These services included the availability of contraceptives, health-facility delivery, pregnancy testing, and ante- and post-natal care. However, the findings on educating early adolescents about birth control methods were mixed, especially injections, pills, and condoms. Participants from FBOs did not recommend education on condoms, and they believed such education would encourage early sexual activity and, therefore, supported sexual abstinence-only education. Nevertheless, participants from public institutions noted the need to introduce and educate about birth control methods to prevent early pregnancies. However, a participant from a public institution neither did support the idea of availing condoms to early adolescents nor perceived it as a top priority (Table 2).

The second area of CSE mainly covered by the curriculum in Rwanda was the human body and development (key concept 6). This area comprises four topics: sexual and reproductive anatomy and physiology, reproduction, puberty and body image [24]. The review of the curriculum yielded, in total, 11 CSE competences in this area. Most of these competences fall under two topics of sexual and reproductive health, anatomy and physiology and puberty (four competences for each). The remaining three competences address to the topic of reproduction, and there were no CSE competences related to body image (Fig. 1). Qualitative findings revealed that CSE for early adolescents focuses on anatomy and physiology, changes, and stages of sexual development. Participants indicated that emphasising these topics allows early adolescents to be aware of physiological changes, related sexual risks and their prevention. In addition, one participant suggested that the competence-based curriculum should consider providing positive information about sex, including enjoyment of sex, rather than focusing merely on its negative consequences (Table 2).

The third area mainly addressed by the competence-based curriculum in Rwanda is values, rights, culture and sexuality. This area comprises three topics: values and sexuality; human rights and sexuality; and culture, society and sexuality [24]. The curriculum review yielded nine CSE competences for early adolescents. Most of these competences (six out of nine) fell under human rights and sexuality, and there were no CSE competences on culture, society, and sexuality (Fig. 1). Qualitative findings in this area indicate that sexuality education for early adolescents should focus on cultural values and norms of “ideal girls” (called *Ni Nyampinga*) who abstain from sex, prevent HIV and other STIs, and/or use condoms. Participants perceived abstinence from sex as an important biblical spiritual requirement. They highlighted the role of FBOs in teaching girls about their specific dress codes, including avoiding miniskirts and trousers and keeping their hair natural. Participants also explored meanings and perceptions associated with dress codes in the context of values, culture and sexuality, saying that the way someone (a girl) dresses has a meaning. They had observed that people associated wearing short clothes like miniskirts with being modern and civilised, which they believed was a wrong perception. On the other hand, participants said that people believed short clothes symbolised attracting people’s attention and a sign of being easily approachable for dating. Finally, participants articulated how the law protects early adolescents, highlighting that adolescents should not be abused and report sexual abuse (Table 2).

The fourth area mainly emphasised by the CSE for early adolescents in Rwanda is understanding gender (key concept 3). This area comprises three topics: social construction of gender and gender norms; gender equality, stereotypes and bias; and gender-based violence [24]. There were eight CSE competences in the curriculum, six of which focused on gender equality, stereotypes, and bias (Fig. 1). Qualitative results in this area revealed that sexuality education for early adolescents focused on gender equality and the importance of giving equal opportunities to both boys and girls. In addition, key informants highlighted that CSE of early adolescents tackled gender roles and responsibilities of boys and girls. While some participants viewed the early adolescent boys and girls’ roles as interchangeable and complementary, despite their biological differences, some participants said they teach early adolescents differential roles and responsibilities in marriage. For example, the husband is expected, among other things, to secure shelter, food and clothing for the family, including the wife. In contrast, married women’s responsibilities involve respecting their husbands and food and bed preparation, including bed making and being ready for sex. Some participants advocated for sexuality education that strengthens self-confidence, decision-making, critical thinking, independence, and self-reliance. At the same time, someone recommended introducing content on lesbian, gay, bisexual, transgender, and intersex (LGBTI) as an integral part of sexuality at later stages of their sexual development in adolescence (Table 2).

The area of relationships (key concept 1) comprises four topics: families; friendship, love and romantic relationships; tolerance, inclusion and respect; and long-term commitment and parenting [24]. The curriculum review showed that this area yielded six CSE competences, most of which fell on friendship, love, and romantic relationships .There were no CSE competences related to long-term commitments and parenting (Fig. 1). Qualitative findings in this area indicated that teaching CSE focused on establishing good and safe relationships with friends, especially between boys and girls. In addition, they suggested that early adolescents should learn about marriage and the qualities of good husbands and wives who will build good families. Key informants from faith-based organisations (FBOs) offered a special emphasis on teaching about long-term commitments, quoting the importance of Biblical virtues, including avoiding conjugal infidelity and abortion, decision-making and the meaning of marriage (Table 2).

The next area of CSE covered by the competence-based curriculum in Rwanda was sexuality and sexual behaviours (key concept 7). This area consists of two topics: sex, sexuality, and sexuality life cycle, and sexual behaviour and sexual response [24]. The curriculum review indicated that this area has only five CSE competences for early adolescents (Fig. 1), mainly on the topic of sexual behaviour and sexual response, which had four competences. Qualitative findings in this area indicated that sexuality education for early adolescents emphasised behaviours that were appropriate to their developmental stage and the consequences of inappropriate behaviours. One participant from a public institution indicated that sexual instinct is an important topic for early adolescents and perceived it as a natural phenomenon of developmental processes but regretted that most teachers and parents tend to overlook it (Table 2).

The area of skills for health and wellbeing (key concept 5) comprises the following five topics: norms and peer influence on sexual behaviours; decision-making; communication, refusal and negotiation skills; media literacy and sexuality; and finding help and support [24]. The curriculum review showed that this area had four CSE competences in total, with three on decision-making and one on norms and peer influence. There were no CSE competences for the remaining three topics (Fig. 1). Qualitative findings in this area highlighted that early adolescents learn to foster their decision-making and skills related to access and use of condoms (Table 2).

The area of violence and staying safe (key concept 4) comprises three topics of violence; consent, violence and bodily integrity; safe use of information and communication technologies (ICTs) [24]. This area was the least represented in CSE competences, with only two competences related to the topic of violence. There were no competences on consent, privacy, bodily integrity, and safe use of information and communication technologies (Fig. 1). Qualitative findings emphasize that early adolescents learn to refuse unwanted sexual activity, resist temptations, and report any information related to sexual violence. In addition, girls learn self-respect, standing firm, and to ‘say no’ to all requests for undesired sexual activity (Table 2).

**Table 2:** Summary of findings from the qualitative study of the content on CSE in Rwanda

Area of CSE	Topics arising from interviews	Illustrative quotes
Good and safe relationships	Safe relationships	*“We do not teach them to be lonely, but we teach them how to behave in good and safe relationships between boys and girls.“* (P07, Male, FBO)
	Importance of Biblical virtues	*“… The bible says that he who has a good wife has a good home. So we start preparing children at their early stages to become good husbands and wives who will start strong families.”* (P11, Male, FBO)
Values, rights, culture and sexuality	Sexual abstinence	*“Teaching a child about not having sex is spiritual because the seventh commandment prohibits us from having unauthorised sex.“* (P11, Male, FBO)
	Dressing norms	*“It is prohibited in our religion to put on trousers, miniskirt, having artificial hairs styles.”* (P03, Female, FBO)
	Prevent sexual abuse	*“The law protects those who are under 18. It is against the law for adults to date a minor. They get arrested.“* (P16, Male, Public Institution)
Understanding gender	Gender equality	*“Gender does not mean favouring boys or girls, but instead giving equal opportunities to all. They [boys and girls] should all be supported the same way and benefit equally from education.”* (P17, Female, Government)
	Gender roles	*“We teach them that there are no roles specific to boys and girls; their roles are interchangeable. We want them to understand that one is capable of doing what another is supposed to do.”* (P01, Female, Ministry)
	Fostering self-confidence	*“We focus on the development of self-confidence, decision-making, and teaching them to have self-trust and how to make informed choices, and critical thinking.”* (P08, Male, FBO)
	Sexual diversity	*“…we know it [LGBTI] is also part of sexuality… I feel the framework around sexuality should incorporate that because it is how they are. Maybe it is not easy to find it among early adolescents, but at 16 to 19 years, you might have cases. So it would be good if programming could also look at this.“* (P02, Female, NGO)
Violence and staying safe	Refusing unwanted sexual activity	*“Sexual and reproductive health education of early adolescents covers … saying NO to unwanted sexual activity, resist temptations and reporting information related to rape.* (P14, Female, Public Institution)
Skills for health and well-being	Independent decision-making	*“We teach them to make informed decisions and their rights, they can get married. They can take decisions without being easily influenced”* (P16, Male, Public Institution)
	Condom use	*“… provide open information on access to condoms. It is very confidential. Girls and boys go and go back and obtain the information they need.”*(P02, Female NGO)
Human body and development	Understanding human body	*“We focus on teaching children about body anatomy and physiology so that they understand how they [their bodies] work… know when it is risky and how they can protect themselves.“* (P16, Male, Public Institution)
	Sexual pleasure	“…*you should also approach the positive side. Boys and girls want to talk about it, they have questions, and sometimes questions are not just the consequences of unprotected sex and early pregnancy but also how can I have pleasurable sex. Sometimes it is very difficult to ignore the positive side of the information.“*
Sexuality and sexual behaviour	Appropriate behaviour	*“They [educators] mostly talk about how early adolescents should behave, and the consequences of inappropriate behaviours.”* (P10, Female, Public Institution)
	Sexual instinct	*“Sexual instinct is something natural which increases as someone grows, but you will not find teachers or parents telling their children about that… but if they were to be taught this earlier to know how to behave in such a situation, to know that sexual intercourse is a natural need”. (P01, Female, Public Institution)*
Sexual and reproductive health	Prevention of pregnancy and STIs	*“Girls do not misbehave like boys. Society does not perceive it as a big deal when boys misbehave. However, it is shameful when girls misbehave. They [adults] fear having girls with an unexpected pregnancy, but boys do not conceive.“* (P03, Female, FBO)
	Circumcision	*“They are taught about circumcision, and I would not say that circumcision is still considered strange as it was before.“* (P13, Female, Public Institution)
	SRH services	*“We focus mainly on access to information. We provide information about services provided regarding reproduction such as contraceptives, going to the hospital for delivery, pregnancy tests, ante- and post-natal care.“* (P16, Male, Public Institution)
	Pros and cons of birth control methods	“*Some [people] believe that teaching contraceptives may encourage [early adolescents] to have sex… sexual activity is already there. Therefore, [we] have a program of teaching [early adolescents] about birth control and different methods they can use.” (P12, Male, FBO)* *“They advise parents to provide condoms to their children when they go to school, but I think that is not the first thing to teach” (P05, Male, Public Institution)*

## Discussion

The current study provides a review of the content of CSE as reflected in the competence-based curriculum for early adolescents in Rwanda, and assesses which aspects of international guidelines on sexuality education are represented in this curriculum of CSE in the Rwandan schools. The literature indicates that sexuality education may be delivered as a stand-alone or integrated subject [7, 25]. In this review, the competence-based curriculum integrates CSE into existing subjects. In other settings, sexuality education is delivered in the subject of Biology to emphasise biological aspects of sexuality [9, 22, 42, 43] rather than focusing on human rights and skills development. As the selection of such subjects depends on the country, the types of school, and other conditions [25, 43], the findings from this study highlighted that the subjects of Social Studies, Science and Elementary Technology, and Knowledge of Environment were the ones that accommodate CSE in Rwanda in addition to other subjects mentioned in the current study.

Integrating CSE into extant subjects is preferred in Sub-Saharan Africa and has the advantage of reducing pressure of an additional subject on students and teachers, as well as the need for a specially trained teacher [44]. However, studies indicate that CSE may be given inadequate weight when integrated into other subjects because it is perceived as diluted into other subjects [45, 46]. In addition, this integration may result in allocating limited budget to CSE for some schools and development of inadequate teaching materials by untrained teachers [22]. Finally, when CSE is integrated into elective and/or non-examinable subjects, students may miss some CSE content, or, some teachers may pay little attention to these subjects that are not examinable [45, 47]. It is however important to note that the competence-based curriculum in Rwanda indicates that the subjects in which CSE is integrated are mandatory and examinable [40]. A standalone CSE subject provides opportunities for monitoring and evaluating programming, and revising the curriculum when necessary [7]. Therefore, delivering CSE as a standalone or integrated subject, or a combination of both in Rwanda has its implications that are beyond the scope of this research.

This study showed that the competence-based curriculum integrates sexuality education in pre-primary education level, which echoes the recommendations to initiate CSE in childhood, including kindergarten and preschool [48]. The number of CSE competences included in the curriculum increases progressively by age, with a small amount of CSE competences in lower grades and high amount of competences in lower-secondary education (12–14 years). This echoes sexuality education guidelines indicate that CSE should be age- and developmentally appropriate [2, 3, 24, 48, 49].

The Government of Rwanda recognizes that CSE should cover equally the eight key areas recommended by ITGSE [34]. However, this review of the competence-based curriculum in Rwanda indicates that CSE emphasises some key concepts and overlooks some equally important others. Of the 58 CSE competences identified through all reviewed CSE curricula, many of them pertained to the key concept of sexual and reproductive health. CSE competences related to understanding, recognising and reducing the risk of STIs, including HIV, and those related to pregnancy and its prevention were respectively the most frequent in the competence-based curriculum. Keogh et al. [30] indicate that imbalances in coverage of CSE are due to prioritisation of certain areas over others. HIV and other STDs and pregnancy prevention were also the main focus of sexuality education programs in the United States of America (USA) [50], which contrasts with current findings. Similarly, in Mexico [51], CSE emphasizes on topics related to the human body and development, such as sexual and reproductive health anatomy, physiology, and puberty. Qualitative findings from the current study showed that the main reason for the Rwandan competence-based curriculum to focus on aspects of reproductive health anatomy and physiology is to teach early adolescents to protect themselves and reduce risks related to unsafe sexual activity, like the sex education in several settings, including the USA [26]. Furthermore, while the participants indicate that CSE focuses on preventing sexual violence among early adolescents, the results from the curriculum review show that this area is the least represented in CSE competences, which suggests a further elaboration of this area in the curriculum.

Although the competence-based curriculum defines the CSE content for early adolescents in Rwanda, it is important to provide a reference book for teachers of the pre-primary and primary levels in order to standardize and optimize the implementation of CSE program in Rwanda. The Rwanda Education Board published in 2018 a teachers’ reference book on CSE in secondary education level [34]. The main objectives of this book are, among others, to help teachers address their own value conflicts and build learners’ knowledge, skills and attitudes related to sexuality, reproductive health and communication. However, this book does not allocate the content to each intended education levels nor provide guidance on CSE for the pre-primary and primary levels, which suggest different pathways of implementation at these levels.

Globally, despite the existence of sexuality education standards [24–27, 52], the content of CSE programs is dissimilar across countries [50]. For example, contrary to Ghana, where most of sexuality topics are taught [30], this review of the competence-based curriculum shows that prescribed contents of CSE programme for early adolescents in Rwanda does not address all key concepts and topics suggested by UNESCO’s ITGSE [24]. This study aligns with the findings from other settings that there are differences and gaps in sexual health education curriculum content within and across countries, namely in the USA, United Kingdom, Mainland China, Hong Kong, and Taiwan [3]. While sexual health education in Kenya puts little emphasis on gender and sexual and reproductive rights [30], this study indicated the need to emphasise on the key concepts that were not sufficiently reflected in the CSE programme for early adolescents in Rwanda. These key concepts include violence and staying safe (key concept 4), skills for health and wellbeing (key concept 5), sexuality and sexual behaviours (key concept 7), and relationships (key concept 1). In addition, these key concepts have a limited number of CSE competences in the competence-based curriculum. In addition, eight of the 27 CSE topics suggested by UNESCO’s ITGSE [24] are not addressed by the CSE programme for early adolescents as indicated in this study. These findings suggest that early adolescents officially receive little or no information about the following important topics: long-term commitment and parenting; cultural aspects of sexuality; bodily integrity; safe use of information and communication; negotiation of safe sex; effective use of media for better sexual health, seeking help and body image. Furthermore, six recommended CSE topics appear only once in the competence-based curriculum in Rwanda, which suggests a further representation of these topics in the curriculum. These topics include families; and tolerance, inclusion, and respect in key concept 1; social construction of gender and gender norms and gender-based violence in key concept 3; norms and peer influence on sexual behaviour in key concept 5; and sex, sexuality and sexual life cycle in key concept 7.

Qualitative findings suggested additional CSE topics to the competence-based curriculum in Rwanda, such as safe sex, sexual pleasure, desire, and sexual orientation. This study noted that the CSE curriculum tends to focus more on the consequences of unprotected sexual activity and fails to consider safer and pleasurable sex topics. Despite being controversial [9], a previous qualitative study indicates that discourse around pleasured sex is often associated with negative outcomes such as not using condoms, rushing into sex, regretted sex, pregnancy or sexually transmitted diseases [53]. However, many scholars advocate that this topic be included in CSE content [9, 17, 26, 49, 54], as CSE on sexual expression and pleasure yielded more positive outcomes [16]. Integrating sexual pleasure into CSE offers a positive approach to adolescents’ sexuality, leading to increased contraceptive use and sexual agency [43].

Although mentioned by only one participant in this study, teaching young adolescents about controversial topics such as sexual orientation, namely about lesbian, gay, bisexual, transgender, or intersex (LGBTI), is an important topic that aligns with international guidance on sexuality education [24]. This finding echoes the recommendations from Crockett, Deardorff [42] to prevent associated harassment and discrimination against LGBTI people. In this perspective, scholars in Sub-Saharan Africa advocate for including in the curriculum and teaching culturally controversial topics such as abortion, homosexuality, and masturbation because these topics are avoided, skipped in the CSE programs or discussed in a negative light [44]. According to the UNFPA, sexual diversity is part of the topics about which children and young people should acquire accurate information [23]. In addition, the need to extend CSE to practical skills related to contraceptives was documented in Ghana [55].

Due to anticipating the consequences of an unexpected pregnancy, participants in the current study perceived that CSE for early adolescents focuses on pregnancy prevention more for girls than for boys. A study on young Romanian people indicated that, at the family level, girls get involved most often in sexual and reproductive health education compared to boys to prevent the negative effects of sexual activity, including early pregnancy [56]. In addition, research evidence on gender norms and sexuality of young adolescents in Uganda indicated that, compared to boys, adolescent girls experience more challenges in their sexual health and need more protection and supervision [57]. A previous study depicts girls as more vulnerable to unwanted sexual behaviour [58]. This study found that the competence-based curriculum focuses narrowly on violence and staying safe (key concept 4) and stresses the need to educate early adolescents, especially girls, on this topic, as they are more prone to physical and sexual assault than boys [50].

Participants from the current study disagree about the presence of some topics in the CSE programme for early adolescents in Rwanda such as abstinence, birth control methods including condoms. Participants from FBOs rely on Biblical virtues and support contents encouraging abstinence while public institutions aim to introduce birth control methods to prevent early pregnancy. FBOs argue that some content of CSE, especially condom use, because it compromises moral values and may lead to initiating early sexual activity among early adolescents. Studies indicate that CSE has significant positive results on adolescents’ behaviours [59, 60] and increases condom or contraceptive use [61]. Furthermore, evidence from a cluster randomized trial in Uganda concluded that delivering CSE to early adolescents does not result in early initiation of sexual activity [18].

This study highlights the influence of FBOs on the content of CSE programs and it is important to note that the Rwandan population is highly religious, as 93% of them are affiliated with different religions, dominated by Christianity [37]. Research in east and southern Africa indicates that religious leaders are willing to provide and influence CSE programmes for children in Africa because of the presence of religious institutions in most communities and their authority [62]. It is equally important to note that effective CSE should be broader and provides a wide range of possibilities for young people to practice safer safe, and is not limited to promoting abstinence only [63]. Regrettably, studies on sexuality education in four low- and middle-income countries highlight that contraception methods are neglected [30] or less emphasised [9, 64], and CSE focuses only on abstinence as the main method of preventing pregnancy [44]. The ITGSE suggests that engaging with religious leaders may help resolve these different views on the content of CSE programmes and conciliate what religions teach, scientific evidence, and lived reality of young adolescents [24].

### Limitations

We do not claim to having exhausted the analysis of all topics covered by comprehensive sexuality education as reflected in the competence-based curriculum in Rwanda. This study reviews only the competence-based curriculum published by the Rwanda Education Board, the only institution mandated to develop and distribute curricula documents to Rwandan public schools. Private international schools’ curricula were not included in this review. Despite this limitation, this study provides an overview of the scope of CSE programme for early adolescents for safe sexual and reproductive health and the extent to which the competence-based curriculum aligns with international guidelines on sexuality education. Further studies would assess the quality and challenges related to implementing CSE among early adolescents. There is also a need to extend this study to early out-of-school adolescents. Furthermore, this review was limited to the curriculum designed for public schools although some private schools use the same curricular. Future research would also aim to understand how private schools using other international curricula in Rwanda meet the education need of early adolescents in terms of CSE coverage, which would provide additional findings to reflect on the overall content of CSE for early adolescents in general. Finally, there is a need to understand how the implementation of the Rwandan competence-based curriculum influences learning about CSE for early adolescents. Specifically, studies should assess whether CSE competences described in the curriculum correspond to what early adolescents really learn in their schools or represent their actual CSE competences in Rwanda, as well as what factors influence the implementation of CSE curriculum in Rwanda.

## Conclusion

CSE is an effective means to facilitate access to sexual and reproductive health and rights information and improve the knowledge of young adolescents. The Rwandan competence-based curriculum introduced the CSE to respond to inadequate knowledge about sexuality, gender, and reproductive health among learners in 2016. The review of this curriculum suggests that it aligns to some extent with international guidelines on sexuality education but needs further elaboration to serve its purpose among early adolescents in Rwanda. In addition, education stakeholders in Rwanda need to harmonize the content of CSE and extend the focus to areas of CSE that are not adequately addressed, including those that are still locally controversial but internationally recommended.

## Data Availability

The datasets used and/or analysed during the current study are available from the corresponding author on reasonable request.

## Abbreviations

CARTA: Consortium for Advanced Research Training in Africa
CMHS: College of Medicine and Health Sciences
CSE: Comprehensive Sexuality Education
ITGSE: International Technical Guidance on Sexuality Education. IRB:Institutional Review Board
HREC: Human Research Ethics Committee
NCST: National Council for Science and Technology
REB: Rwanda Education Board
SRH: Sexual and reproductive health
SRHR: sexual and reproductive health and rights
